# Identification of Neoantigens in Two Murine Gastric Cancer Cell Lines Leading to the Neoantigen-Based Immunotherapy

**DOI:** 10.3390/cancers14010106

**Published:** 2021-12-27

**Authors:** Koji Nagaoka, Changbo Sun, Yukari Kobayashi, Takayuki Kanaseki, Serina Tokita, Toshihiro Komatsu, Kazuhiro Maejima, Junichiro Futami, Sachiyo Nomura, Keiko Udaka, Hidewaki Nakagawa, Toshihiko Torigoe, Kazuhiro Kakimi

**Affiliations:** 1 Department of Immunotherapeutics, The University of Tokyo Hospital, Tokyo 113-8655, Japan; knagaoka@m.u-tokyo.ac.jp (K.N.); sonc-sur@h.u-tokyo.ac.jp (C.S.); yukkoba@m.u-tokyo.ac.jp (Y.K.); 2 Department of Pathology, Sapporo Medical University, Sapporo 060-8556, Japan; kanaseki@sapmed.ac.jp (T.K.); st.tokita@gmail.com (S.T.); torigoe@sapmed.ac.jp (T.T.); 3 Sapporo Dohto Hospital, Sapporo 065-0017, Japan; 4 Department of Immunology, Kochi University, Kochi 783-8505, Japan; tkomatsu@kochi-u.ac.jp (T.K.); udaka@kochi-u.ac.jp (K.U.); 5 RIKEN Center for Integrative Medical Sciences, Laboratory for Cancer Genomics, Yokohama 230-0045, Japan; kazuhiro.maejima@riken.jp (K.M.); hidewaki@riken.jp (H.N.); 6 Department of Interdisciplinary Science and Engineering in Health Systems, Okayama University, Okayama 700-8530, Japan; futamij@okayama-u.ac.jp; 7 Department of Gastrointestinal Surgery, Graduate School of Medicine, The University of Tokyo, Tokyo 113-8655, Japan; snomura-gi@umin.ac.jp

**Keywords:** gastric cancer, neoantigen, checkpoint inhibitor, DC vaccine, adoptive cell therapy (ACT)

## Abstract

**Simple Summary:**

Despite the success of immune checkpoint inhibitors (ICI) for treating a variety of solid cancers, most gastric cancer patients are resistant to ICI monotherapies. Combinations of ICI with other therapies may be able to overcome this resistance. In order to develop combination immunotherapies, immunologically well-characterized preclinical gastric cancer models are required. To this end, in the present study, we characterized two murine gastric cancer cell lines, namely, YTN2 which spontaneously regresses, and YTN16 which grows progressively. Although anti-CTLA-4 monotherapy eradicated most YTN16 tumors, these were resistant to either anti-PD-1 or anti-PD-L1 treatment. Furthermore, we identified neoantigens in YTN2 and YTN16 tumors and conducted neoantigen-based immunotherapy for these tumors. In addition, the information on neoantigens facilitates the evaluation of tumor-specific immune responses induced by the combination therapies. These immunologically well-characterized gastric cancer models will contribute to the development of novel combination immunotherapies.

**Abstract:**

To develop combination immunotherapies for gastric cancers, immunologically well-characterized preclinical models are crucial. Here, we leveraged two transplantable murine gastric cancer cell lines, YTN2 and YTN16, derived from the same parental line but differing in their susceptibility to immune rejection. We established their differential sensitivity to immune checkpoint inhibitors (ICI) and identified neoantigens. Although anti-CTLA-4 mAbs eradicated YTN16 tumors in 4 of 5 mice, anti-PD-1 and anti-PD-L1 mAbs failed to eradicate YTN16 tumors. Using whole-exome and RNA sequencing, we identified two and three neoantigens in YTN2 and YTN16, respectively. MHC class I ligandome analysis detected the expression of only one of these neoantigens, mutated Cdt1, but the exact length of MHC binding peptide was determined. Dendritic cell vaccine loaded with neoepitope peptides and adoptive transfer of neoantigen-specific CD8^+^ T cells successfully inhibited the YTN16 tumor growth. Targeting mutated Cdt1 had better efficacy for controlling the tumor. Therefore, mutated Cdt1 was the dominant neoantigen in these tumor cells. More mCdt1 peptides were bound to MHC class I and presented on YTN2 surface than YTN16. This might be one of the reasons why YTN2 was rejected while YTN16 grew in immune-competent mice.

## 1. Introduction

Gastric cancer is the fifth most frequent cancer and the fourth most common cause of cancer death worldwide [1]. Anti-PD-1 checkpoint blockade therapies, such as those employing treatment with the monoclonal antibodies pembrolizumab and nivolumab, have been trialed for treating advanced gastric cancer [2,3]. Although pembrolizumab monotherapy has demonstrated anti-tumor activity in that small proportion of patients with metastatic gastric cancers having high microsatellite instability and Epstein–Barr virus-positivity [4], the majority of patients with more common forms of gastric cancer is resistant to such monotherapy. A combination of immune checkpoint inhibitors (ICI) with other therapies is therefore being considered as an approach to overcome resistance. Many clinical trials are ongoing or have reached their endpoints, and recently, the combination of nivolumab plus chemotherapy has been approved by the FDA [5]. However, the anti-tumor effect is still not sufficient to cover all patients and the development of additional combination therapies remains an urgent need.

For developing targeted therapies for cancer, it is common for candidate drugs to be initially evaluated using cancer cell lines in culture and xenografts in immunodeficient mice. Given that ICI therapy targets the host’s immune system, immunocompetent animal models are needed to facilitate the development of new combination therapies. Because no immunologically well-characterized transplantable murine gastric cancer cell lines were available, from one tumor we established four subclones that could be transplanted into C57BL/6 mice [6]. When inoculated subcutaneously, two of these, YTN2 and YTN3, were found to be less aggressive than two others designated YTN5 and YTN16, which were very aggressive. We found that the most aggressive subclone YTN16 expressed FGFR4 at high levels and that disruption or inhibition of FGFR4 suppressed tumor growth both in a subcutaneous and peritoneal dissemination model. Furthermore, based on the infiltration of IL-17-producing T cells into YTN16 tumors, a combination of anti-PD-1 and anti-IL-17 mAb treatment was tested and found to suppress YTN16 tumor growth [7]. These results indicated that these gastric cancer cell lines could be utilized for the development of molecular targeted therapy.

These earlier studies demonstrated that these gastric cancer cell lines might also be valuable for developing cancer immunotherapies. To this end, here we characterize two of these gastric cancer cell lines, YTN2 and YTN16, in terms of the expression of neoantigens derived from tumor-specific mutant proteins. Our findings may provide useful information for enhancing the development of models for novel effective immunotherapies for gastric cancers.

## 2. Materials and Methods

### 2.1. Mice, Tumor Cells and Reagents

Six-week-old female C57BL/6N mice were purchased from Japan SLC (Shizuoka, Japan) and kept in a specific pathogen-free environment. The animal use proposal and experimental protocols were reviewed and approved by The University of Tokyo Animal Care and Use Committee (ID:P15-125) and all animal procedures were conducted in accordance with institutional guidelines. The YTN2 and YTN16 cell lines [6] were maintained in Dulbecco’s modified Eagle’s medium (DMEM, Nacalai Tesque, Kyoto, Japan) with 10% heat-inactivated fetal bovine serum (FBS, Sigma-Aldrich, St. Louis, MO, USA), 100 μg/mL streptomycin, 100 U/mL penicillin (Nacalai Tesque), and MITO+ serum extender (Corning, Corning, NY, USA). Anti-PD-1 (RMP1-14), PD-L1 (10F9G2), CTLA-4 (9H10), and CD8α (53–6.7) mAbs were from Bio X Cell (Lebanon, NH, USA). DimerXI:Recombinant Soluble Dimeric Mouse H-2D^b^:Ig (H-2D^b^ dimer), DimerXI:Recombinant Soluble Dimeric Mouse H-2K^b^:Ig (H-2K^b^ dimer) were from BD Biosciences (Franklin Lakes, NJ, USA). FITC-conjugated anti-CD3ε, PerCP/Cyanine5.5-conjugated anti-CD4, APC/Cyanine7-conjugated anti-CD8, APC-conjugated anti-IFN-γ and Pacific Blue-conjugated anti-CD45 mAbs were from BioLegend (San Diego, CA, USA).

### 2.2. Transplantation of YTN2 and YTN16

Mice were inoculated with 5 × 10^6^ YTN2 or YTN16 subcutaneously into the right flank. Anti-PD-1 (200 μg), anti-PD-L1 (200 μg), anti-CTLA-4 (100 μg), and/or anti-CD8α (200 μg) were injected intraperitoneally. Tumor growth was monitored every 2 to 3 days with calipers, and tumor volume was calculated by the formula π/6 × *L*_1_*L*_2_*H*, where *L*_1_ is the long diameter, *L*_2_ is the short diameter, and *H* is the height of the tumor.

### 2.3. Whole-Exome Sequencing (WES)

Genomic DNA was extracted from YTN2, YTN16, LLC1, and B16F10 cell lines using Allprep DNA/RNA mini kits (Qiagen, Venlo, The Netherlands) according to the manufacturer´s protocols. DNA was randomly fragmented by Covaris and adapters were ligated to both ends of the fragments. DNA was then amplified by ligation-mediated PCR, purified, and hybridized to the Roche NimbleGen SeqCap EZ Exome probe (Roche, Basel, Switzerland). The captured library was loaded onto the HiSeq 2000 and HiSeq 4000 platforms (Illumina, San Diego, CA, USA). After trimming of adapter sequences, reads were aligned to mm10 mouse reference sequences using the Burrows–Wheeler Aligner (BWA). Somatic variants were detected using SOAPsnp. Raw data were deposited in the Sequence Read Archive (SRA) database (accession number SRR12072973-75, SRR15647456 and SRR 17087999). WES data of MC38 was downloaded from the SRA database (SRR5684459).

### 2.4. RNA Sequencing (RNA-Seq)

Total RNA was isolated using TRIzol RNA Isolation Reagent (Thermo Fisher Scientific, Waltham, MT, USA) and RNeasy mini kits (Qiagen). RNA-Seq libraries were prepared using TruSeq Stranded mRNA Library Prep (Illumina) according to the manufacturer’s protocols and sequenced on NovaSeq 6000 and HiSeq X systems (Illumina). The reads were aligned to mm10 reference sequences with STAR (version 2.7.0f). The mapped reads were counted with featureCounts (version 1.6.4). Fragments per kilobase of exon per million reads mapped (FPKM) was calculated by the formula Y/LN × 10^9^, where Y is the number of fragments mapped to a gene; L is the length of the gene; N is the total number of mapped reads of a sample. Raw data were deposited in the Gene Expression Omnibus (GEO) database (GSE146027 and GSE184092).

### 2.5. Epitope Prediction

First, we selected expressed mutations as follows: FPKM ≥ 30 and RNA variant allele frequency (VAF) ≥ 0.04. Then 8-, 9-, and 10-mer epitopes containing the mutated amino acid were inspected using NetMHCpan2.8 [8] and NetMHCpan4.1 [9] for prediction of IC_50_ and eluted ligand (EL) rank to H-2D^b^ and H-2K^b^. Presentation percentiles of MHCflurry (ver.2.0.1) [10] were predicted using 21-mer sequences with the mutated amino acid in the middle. Epitopes with IC_50_ of NetMHCpan ≤ 250 nM, EL rank of NetMHCpan ≤ 0.5 and presentation percentile of MHCflurry ≤ 0.5 were selected. Peptides were synthesized by standard solid-phase synthesis using a Syro I (Biotage, Uppsala, Sweden) as described previously [7].

### 2.6. MHC Class I Stabilization Assay

Peptide binding was measured by a stabilization assay using TAP-deficient RMAS cells [11,12]. Briefly, 1 × 10^5^ RMAS cells incubated overnight at 26 °C were mixed with titrated peptide concentrations in 0.25% BSA-containing DMEM, incubated for 30 min at room temperature, and then exposed to 37 °C for 70 min. Cells were then stained with FITC-labeled anti-K^b^ mAb (B8.24.3) or FITC-labeled anti-D^b^ mAb (B22.249) and analyzed by flow cytometry. Differences between experiments were normalized by including the reference peptides in every assay.

### 2.7. MHC Class I Ligandome Analysis for Neoantigen Identification

Direct detection of neoantigens using mass spectrometry has been described previously [13]. Briefly, frozen cell pellets of 1.5 × 10^9^ YTN2 or YTN16 cells were lysed in buffer containing 0.25% sodium deoxycholate, 0.2 mM iodoacetamide, 1 mM EDTA protease inhibitor cocktail (Sigma-Aldrich), 1 mM PMSF, and 1% octyl-β-D glucopyranoside (Dojindo, Kumamoto, Japan). The peptide-MHC class I complexes were captured by affinity chromatography using purified monoclonal antibodies (clone Y-3 for K^b^ and 28-14-8S for Db) coupled to CNBr-activated Sepharose 4B (GE Healthcare, Chicago, IL, USA). Peptides bound to MHC class I were eluted with a mild acid (0.2% TFA) and desalted using a Sep-Pak C18 cartridge (Waters Corporation, Milford, MA, USA) with 28% ACN in 0.1% TFA and ZipTip U-C18 (Merck Millipore, Burlington, MA, USA) with 50% ACN in 1% FA. Samples were dried using vacuum centrifugation and dissolved in 5% ACN in 0.1% TFA for LC-MS/MS analysis. Samples were loaded into a nano-flow LC (Easy-nLC 1000 system, Thermo Fisher Scientific) online-coupled to an Orbitrap mass spectrometer equipped with a nanospray ion source (Q Exactive Plus, Thermo Fisher Scientific). The nonsynonymous mutations unique to YTN2 or YTN16 were translated in frame and the polypeptide sequences encompassing mutations with a maximum length of 61-mer amino acids were matched against a conventional protein reference database (Swiss-Prot). MS/MS data were searched against personalized custom reference databases using Sequest HT and Mascot (Matrix Science, Boston, MA, USA) on the Proteome Discoverer platform (Thermo Fisher Scientific) with a tolerance of precursor and fragment ions of 10 ppm and 0.02 Da, respectively. A false discovery rate (FDR) of 0.01 was used in the Percolator node of the Proteome Discoverer version 2.2 software (Thermo Fisher Scientific) as the peptide detection threshold.

### 2.8. Mild Acid Elution of MHC Class I-Binding Peptides

MHC class I binding peptides were eluted from viable YTN16 cells as described previously [14]. Firstly, 1.5 × 10^8^ IFN-γ-treated (10 U/mL, PeproTech, Cranbury, NJ, USA) YTN16 cells were treated with citrate-phosphate buffers (0.131 M citric acid/0.066 M Na_2_HPO_4_, pH 3.3) for 1 min at room temperature. The eluted peptides were loaded onto a Sep-Pac C18 cartridge (Waters Corporation), washed with water, eluted with 60% acetonitrile, lyophilized using a Solvent SpeedVac (Thermo Fisher Scientific), and reconstituted in the citrate-phosphate buffer. Peptides of 3000 Da were then isolated by filtration through Centricon-3 ultrafiltration devices (Merck Millipore). The resulting flowthrough was then fractionated by reverse-phase high-performance liquid chromatography (HPLC). Briefly, bulk peptides were fractionated on a YMC-Pack ODS-A column (YMC, Kyoto, Japan) by the following gradient from solvent A (1% acetonitrile/0.1% HCl) to solvent B (80% acetonitrile/0.1% HCl): from 0 to 5 min, 0% B; from 5 to 10 min 0–12.5% B; from 10 to 70 min, 12.5–75% B. The flow rate was maintained at 0.5 mL/min, and fractions (0.5 mL each) were collected from 15 min to 70 min. The fractions were lyophilized. For T cell assays, the lyophilized samples were reconstituted in 50 μL HBSS and 10 μL were added to 1 × 10^5^ cells from the mutated (m) Zfp106-reactive CD8^+^ T cell line in 200 μL medium. After overnight culture, the supernatants were harvested and IFN-γ was evaluated using Mouse IFN-γ ELISA Ready-SET-Go kit (Thermo Fisher Scientific) according to the manufacturer’s protocol.

### 2.9. Screening of Candidate Neoepitope Peptides

To generate YTN16 cell line-specific CD8^+^ T cell lines, YTN16 tumor-bearing mice were treated with anti-PD-1 and/or anti-CTLA-4 mAbs. The spleens were harvested from mice that rejected the tumor, and 5 × 10^6^ splenocytes were stimulated with IFN-γ (10 U/mL) and 1 × 10^5^ irradiated (100 Gy) cells from the YTN16 cell line in the presence of 100 U/mL recombinant human IL-2 (Novartis Corporation, Basel, Switzerland). The cells were re-stimulated with YTN16 on day 7 and harvested on day 12–14. They were screened for reactivity to YTN16 antigens by separately culturing them (1 × 10^5^) overnight with 11 candidate short peptides at 1 μg/mL, harvesting the supernatants, and quantifying IFN-γ production by ELISA.

### 2.10. Establishment of Neoantigen-Specific CD8^+^ T Cell Lines

For establishment of the mZfp106-reactive CD8^+^ T cell line, splenocytes from mice that had rejected YTN2 were stimulated with IFN-γ-treated (10 U/mL) and 100 Gy-irradiated YTN2 cells four times weekly. For establishment of the mCdt1-reactive CD8^+^ T cell line, splenocytes of mice that had rejected YTN16 following treatment with anti-CTLA-4 mAb were stimulated with the 9-mer mCdt1 peptide at 1 μg/mL four times weekly. For establishment of the mScarb2-reactive CD8^+^ T cell line, splenocytes of mice that had rejected YTN16 after anti-CTLA-4 treatment were stimulated with IFN-γ-treated (10 U/mL) and 100 Gy-irradiated YTN16 cells. Seven days later, mScarb2-H-2D^b^ dimer^+^ CD8^+^ cells were sorted on an SH800S (Sony Corporation, Tokyo, Japan) and stimulated with IFN-γ-treated (10 U/mL) and 100 Gy-irradiated YTN16 cells another three times weekly.

### 2.11. Cell Preparation and Flow Cytometry

Tumors were cut into pieces and incubated in RPMI-1640 (Nacalai Tesque) supplemented with 1% FBS, 10 mM HEPES, 0.2% collagenase (FUJIFILM Wako Pure Chemical Corporation, Osaka, Japan) and 2 KU/mL DNase I (Sigma-Aldrich) for 40 min at 37 °C. All material was passed through a 70 μm cell strainer to obtain single cell suspensions. After staining dead cells using a Zombie Yellow Fixable Viability Kit (BioLegend) and blocking of Fc receptors with anti-CD16/32 mAb (2.4G2, Bio X Cell), the cells were stained with mAbs for cell surface antigens, H-2D^b^ and H-2K^b^ dimers. For intracellular cytokine staining, 1 × 10^5^ cells were stimulated with peptides, 1 × 10^5^ YTN2 or 1 × 10^5^ YTN16 cells in the presence of 10 μg/mL brefeldin A (Sigma-Aldrich) for 4 h. After staining dead cells with the Fixable Viability Dye eFluor 450 (Thermo Fisher Scientific) and blocking Fc receptors with anti-CD16/32 mAb, the cells were stained with mAbs for cell surface antigens, followed by fixation, permeabilization, and staining with APC-conjugated anti-IFN-γ mAb (BioLegend) using Intracellular Staining Fixation Buffer and Intracellular Staining Permeabilization Wash Buffer (10X) (BioLegend) according to the manufacturer’s protocols.

### 2.12. Dendritic Cell (DC) Vaccine

DCs were prepared as described previously [15]. Briefly, bone marrow cells from femurs and tibias were cultured in RPMI-1640 supplemented with 10% FBS, 10 mM HEPES (Nacalai Tesque), 1 mM sodium pyruvate (Nacalai Tesque), MEM Non-Essential Amino Acids Solution (Nacalai Tesque), 5 mM 2-mercaptoethanol (Sigma-Aldrich), 100 U/mL penicillin, 100 μg/mL streptomycin, and 20 ng/mL GM-CSF (PeproTech) for 8 days. DCs were stimulated with 1 μg/mL lipopolysaccharide (FUJIFILM Wako Pure Chemical Corporation) for 16 h and pulsed with peptides at 1 μg/mL for 2 h. For induction of neoantigen-specific CD8^+^ T cells, 1 × 10^6^ neoepitope peptide-pulsed DCs were subcutaneously injected into the flank of mice twice biweekly. Two weeks after the second vaccination, the splenocytes were stimulated with the corresponding peptides and IFN-γ production was determined by intracellular cytokine staining. For evaluation of anti-tumor effects of neoepitope peptide-pulsed DC vaccines, mice were inoculated with 5 × 10^6^ YTN16 cells on day 0 into the right flank. Neoepitope peptide-pulsed DCs were injected into the left flank on day 5. Tumor growth was monitored every 2 to 3 days.

### 2.13. Adoptive Transfer of Neoantigen-Reactive CD8^+^ T Cells

Mice were inoculated with 5 × 10^6^ YTN16 cells on day 0. On day 6, 1 × 10^7^ neoantigen-reactive CD8^+^ T cells were infused intravenously. Tumor growth was monitored every 2 to 3 days.

### 2.14. Statistical Analysis

Data are presented as mean ± SD. Statistical analyses were performed with Prism software (version 9.1.0, GraphPad Software, LLC, San Diego, CA, USA). Comparison of results was carried out by one-way ANOVA testing with Dunnet’s multiple comparison test.

## 3. Results

### 3.1. Comparison of Intratumoral Immune Responses to Gastric Cancer Cell Lines YTN2 and YTN16

**Figure 1:**
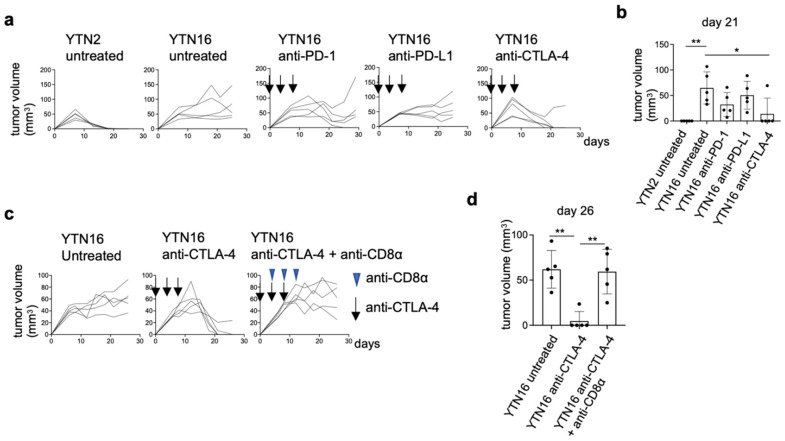
Immunological characterization of YTN2 and YTN16 in terms of tumor growth and sensitivity to checkpoint inhibitors: (**a**) Mice (*n* = 5) were inoculated with 5 × 10^6^ YTN2 or YTN16 cells on day 0. Anti-PD-1 (clone RMP1-14, 200 µg/mouse), anti-PD-L1 (clone 10F9G2, 200 µg/mouse) or anti-CTLA-4 (clone 9H10, 100 µg/mouse) mAbs were administered on days 0, 4, and 8. Tumor volumes were monitored every 2 or 3 days. Arrows indicate the days of antibody injection. (**b**) Tumor volumes on day 21 were shown. * *p* < 0.05, ** *p* < 0.01, one-way ANOVA with Dunnett’s test for multiple comparisons. (**c**) Mice (*n* = 5) were inoculated with 5 × 10^6^ YTN16 cells on day 0. Anti-CTLA-4 and anti-CD8α (clone 53-6.7, 200 µg/mouse) mAbs were injected on days 0, 4 and 8, and 4, 8, and 12, respectively. Tumor volumes were monitored every 2 or 3 days. Arrows and arrowheads indicate the days of anti-CTLA-4 and anti-CD8α injection, respectively. (**d**) Tumor volumes on day 26 were shown. ** *p* < 0.01, one-way ANOVA with Dunnett’s test for multiple comparisons.

YTN2 and YTN16 are subclones derived from the same gastric cancer cell line established from an N-methyl-N-nitrosourea (MNU)-induced tumor [6]. Subcutaneously inoculated YTN2 formed a small nodule in the first week and spontaneously regressed in three weeks (Figure 1a,b). In contrast, YTN16 grew slowly but progressively (Figure 1a). As we reported previously, the rejection of YTN2 is CD8^+^ T cell-mediated [7]. Therefore, YTN16 tumor-bearing mice were treated with ICI using anti-PD-1, anti-PD-L1 or anti-CTLA-4 mAbs, to test whether T cell-mediated immunity against YTN16 tumors could be generated. Anti-CTLA-4 treatment resulted in the eradication of tumors in four out of five mice (Figure 1a,b), whereas anti-PD-1 temporarily inhibited YTN16 tumor growth but failed to eradicate the tumor in four out of five mice. As expected, depleting CD8^+^ T cells using anti-CD8α mAb prevented tumor rejection on anti-CTLA-4 antibody treatment (Figure 1c,d), indicating that ICI-mediated rejection of YTN16 was dependent on CD8^+^ T cell responses.

### 3.2. Identification of Neoantigens in YTN2 and YTN16 Tumors

**Figure 2:**
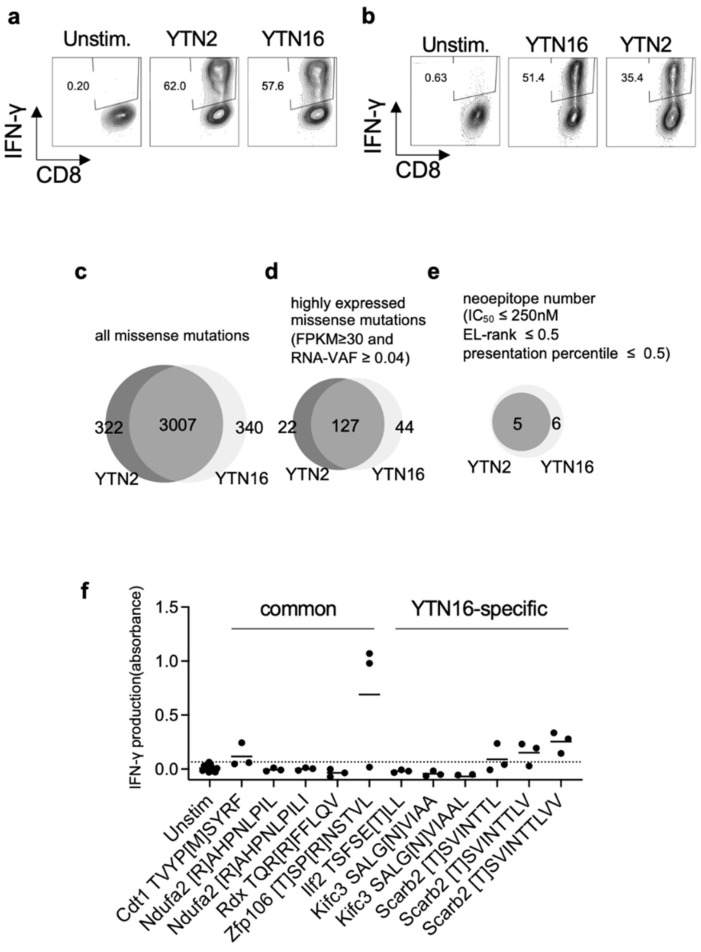
Identification of neoantigens: (**a**) YTN2-reactive CD8^+^ T cell lines were established from the spleens of mice that had rejected YTN2 tumors. They responded to both YTN2 and YTN16 tumor cells and produced IFN-γ. (**b**) YTN16-reactive CD8^+^ T cell lines were established from splenocytes of mice that had rejected YTN16 following treatment with anti-PD-1 and/or anti-CTLA-4 mAbs. IFN-γ production by YTN16-reactive CD8^+^ T cells stimulated with YTN16 or YTN2 was quantified. Venn diagrams indicate (**c**) missense mutations identified by WES, (**d**) expressed mutations filtered by RNA-Seq data (FPKM ≥ 30 and RNA VAF ≥ 0.04), and (**e**) candidate neoepitopes of YTN2 and YTN16 cells. (**f**) YTN16-reactive CD8^+^ T cell lines were stimulated with 11 candidate peptides. IFN-γ production in the culture supernatant was evaluated by ELISA. Data are based on 3 independently established YTN16-reactive CD8^+^ T cell lines. A dotted line indicates mean + 2 SD of IFN-γ production by the unstimulated cells.

To investigate anti-tumor CD8^+^ T cell responses, we determined the specificity of tumor-reactive CD8^+^ T cells in YTN2 and YTN16 tumor-bearing mice. First, we established YTN2-reactive CD8^+^ T cell lines from the spleens of mice that had rejected YTN2 tumors, and YTN16-reactive CD8^+^ T cell lines from mice that had rejected their YTN16 tumors following treatment with anti-PD-1, anti-CTLA-4, or a combination of anti-PD-1 and anti-CTLA-4 mAbs. After two rounds of weekly in vitro stimulation with irradiated tumor cells, we obtained YTN2- and YTN16-reactive CD8^+^ T cell lines (Figure 2a,b). They cross-reacted each other and produced IFN-γ in response to both YTN2 and YTN16 tumor cells.

In parallel, we performed WES of YTN2 and YTN16 tumor cells and identified 3329 and 3347 missense mutations, respectively, 3007 of which were shared by the two variant lines (Figure 2c and Supplementary Tables S1 and S2). The expression of mutated genes was evaluated by RNA-Seq; 149 and 171 mutations were highly expressed in YTN2 and YTN16, respectively, with FPKM ≥ 30 and RNA VAF ≥ 0.04 (Figure 2d and Supplementary Tables S1 and S2). Neoepitopes derived from the products of these mutated genes were predicted by their estimated binding capacity to MHC class I molecules; 8-, 9-, and 10-mer peptides containing the mutated amino acid were assessed by NetMHCpan and MHCflurry algorithms to predict their binding to and presentation on H-2D^b^ and H-2K^b^ molecules. Of these, we selected 11 candidate peptides according to the following criteria: IC_50_ of NetMHCpan ≤ 250 nM; EL rank of NetMHCpan ≤ 0.5; presentation percentile of MHCflurry ≤ 0.5. We then synthesized these 11 candidate peptides. Five represented potential neoantigen epitopes for both YTN2 and YTN16, and six only for YTN16 (Figure 2e and Supplementary Table S3). Some of these peptides were assessed for their binding to MHC class I by a stabilization assay (Supplementary Table S4). In silico-predicted peptides indeed stabilized MHC class I expression on RMAS cells. Next, it was assessed whether these 11 neoepitope peptides were recognized by YTN16-reactive CD8^+^ T cells using IFN-γ production in the culture supernatants measured by ELISA as the readout (Figure 2f). We found that mZfp106, mCdt1 and 8-, 9-, and 10-mers of mScarb2 peptides stimulate YTN16-reactive CD8^+^ T cells. Of these neoantigens, Zfp106 A656T&C659R and Cdt1 T258M were present in both YTN2 and YTN16 tumors, whereas the Scarb2 K426T mutation was detected only in YTN16 (Supplementary Tables S1 and S2).

### 3.3. Neoepitope-Specific CD8^+^ T Cell Lines

**Figure 3:**
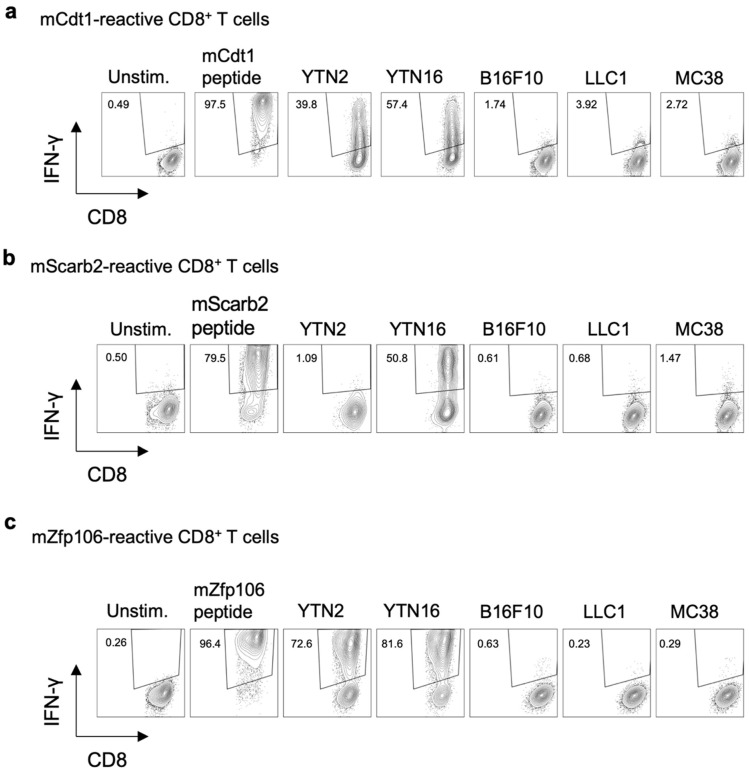
Three neoantigen-reactive CD8^+^ T cell lines. The established neoantigen-reactive CD8^+^ T cell lines were stimulated with the indicated peptides or cells, and their reactivity was evaluated by IFN-γ production: (**a**) mCdt1-reactive CD8^+^ T cells responded to YTN2 and YTN16; they did not respond to B16F10, LLC1 or MC38 cell lines. (**b**) mScarb2-reactive CD8^+^ T cells responded to YTN16; they did not respond to YTN2, B16F10, LLC1, or MC38 cell lines. (**c**) mZfp106-reactive CD8^+^ T cells responded to YTN2 and YTN16; they did not respond to B16F10, LLC1, or MC38 cell lines.

CD8^+^ T cell lines reactive to mCdt1, mScarb2, and mZfp106 were established as follows. For mCdt1-reactive CD8^+^ T cell lines, splenocytes of mice that had rejected YTN16 following treatment with anti-CTLA-4 were repeatedly stimulated with 9-mer mCdt1 peptides (Figure 3a). To establish a mScarb2-reactive CD8^+^ T cell line, splenocytes of mice that had rejected YTN16 after anti-CTLA-4 treatment were stimulated with irradiated YTN16 cells. Seven days later, mScarb2-H-2D^b^-dimer^+^ cells were sorted and expanded by stimulation with irradiated YTN16 cells (Figure 3b). mZfp106-reactive CD8^+^ T cell lines were established by repetitive stimulation of splenocytes from mice that had rejected YTN2 with irradiated YTN2 cells (Figure 3c). These cell lines did not respond to B16F10, LLC1, or MC38 cell lines not containing corresponding mutations (Supplementary Table S5).

### 3.4. Existence of Neoepitope-Reactive CD8^+^ T Cells in YTN2 and YTN16 Tumors

**Figure 4:**
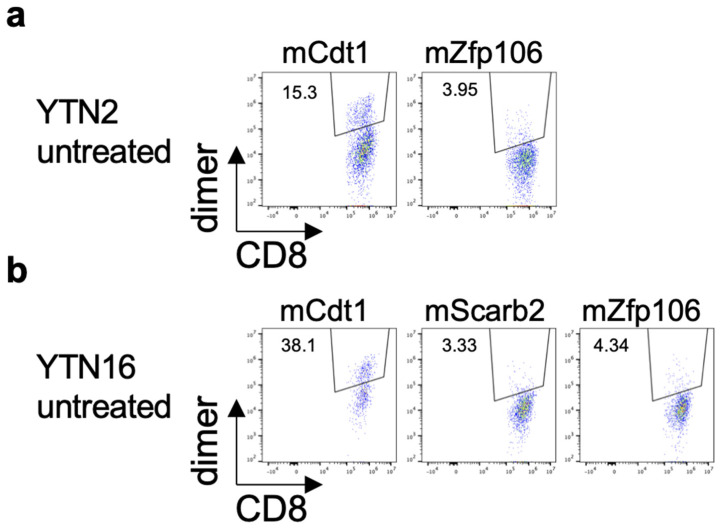
Identification of neoantigen-specific CD8^+^ T cells in the tumor. Mice (*n* = 3) were inoculated with 5 × 10^6^ YTN2 or YTN16 cells on day 0. Tumors were harvested on day 17 and tumor-infiltrating cells were analyzed by flow cytometry. Dot plots show frequencies of mCdt1- and mZfp106-reactive CD8^+^ T cells in YTN2 (**a**) and mCdt1, mScarb2 and mZfp106-reactive CD8^+^ T cells in YTN16 (**b**).

The existence of neoantigen-reactive CD8^+^ T cells in the tumors was investigated by flow cytometry. YTN2 or YTN16 cells were inoculated into C57BL/6 mice on day 0. On day 17, tumor-infiltrating cells were harvested and analyzed by flow cytometry. mCdt1-H-2K^b^-dimer^+^ and mZfp106-H-2D^b^-dimer^+^ CD8^+^ T cells were detected in both YTN2 and YTN16 tumors (Figure 4a,b). mScarb2-H-2D^b^-dimer^+^ CD8^+^ T cells were also detected in YTN16 tumors (Figure 4b).

### 3.5. MHC Class I Ligandome Analysis with Mass Spectrometry

**Figure 5:**
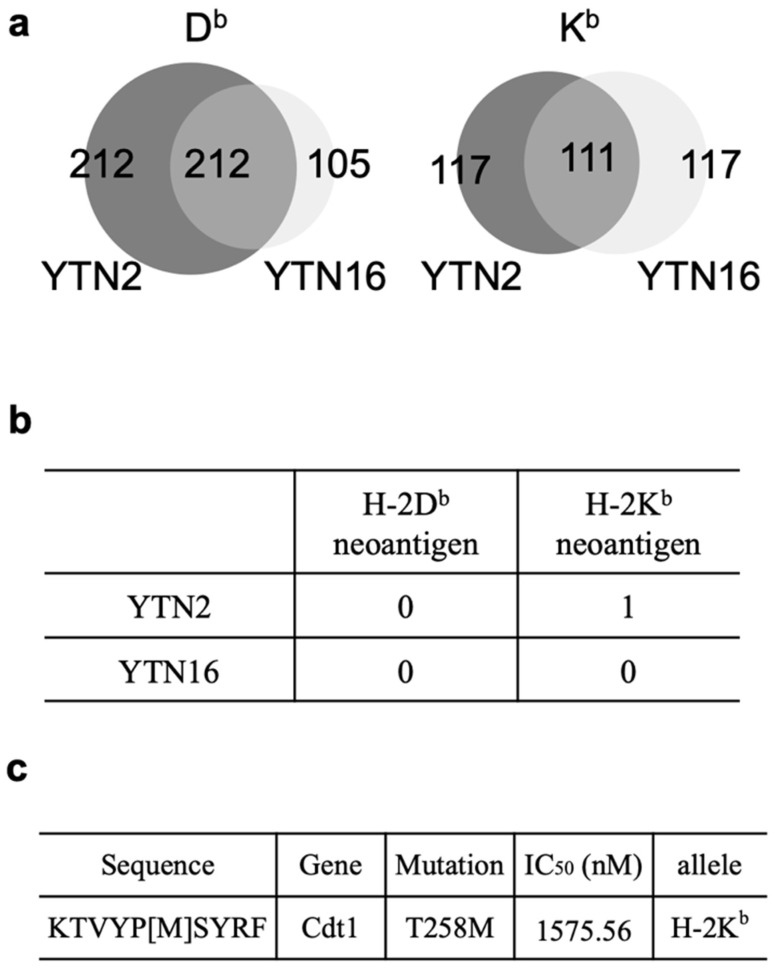
Identification of 10-mer mCdt1 peptides by MHC class I ligandome analysis: (**a**) Venn diagrams indicate the numbers of H-2D^b^- and H-2K^b^-binding peptides identified in YTN2 and YTN16. (**b**) The numbers of H-2D^b^- and H-2K^b^-mutated binding peptides identified in YTN2 and YTN16. (**c**) The amino acid sequence and predicted IC_50_ value of the mCdt1 peptide identified in YTN2.

To directly examine MHC class I-binding peptides in YTN2 and YTN16 tumors, we performed MHC class I ligandome analysis using mass spectrometry. YTN2 and YTN16 cell lines (1.5 × 10^9^ cells) were lysed and peptide-MHC class I complexes were precipitated with anti-MHC class I antibodies. The bound peptides were analyzed by tandem mass spectrometry. From YTN2 tumor cells, 424 H-2D^b^-binding and 228 H-2K^b^-binding peptides were identified (Figure 5a). Although most peptides were wild-type sequences, one of the H-2K^b^-binding eluted peptides was the mCdt1 10-mer peptide KTVYP[M]SYRF (Figure 5b,c). From YTN16 tumor cells, 317 H-2D^b^-binding peptides and 228 H-2K^b^-binding peptides were identified; however, no mutated peptides, also not mCdt1, were detected by ligandome analysis (Figure 5b). In an attempt to determine whether YTN16 cells do present neoepitopes on their surface, we eluted MHC class I-bound peptides from YTN16 cells with mild acid and fractionated them by reverse-phase HPLC (Supplementary Figure S1a). We screened 56 fractionated YTN16 eluates for their ability to stimulate YTN16-reactive CD8^+^ T cells and found that IFN-γ production by mZfp106-reactive CD8^+^ T cells was stimulated by fraction 40 (Supplementary Figure S1b). As mZfp106 peptide had been eluted under the same reverse-phase HPLC conditions (Supplementary Figure S1c), these results indicate that YTN16 tumor cells do present mZfp106 on their surface.

The mRNA expressions of these three neoantigens in YTN2 and YTN16 cells are summarized in Table 1. The FPKM values do not discriminate the expression of wild-type and mutant genes; VAF should be incorporated to evaluate the mutated gene expression. mCdt1 was highly expressed in YTN2 and YTN16; both FPKM and VAF were higher in YTN2 than YTN16. The VAF of mScarb2 in YTN2 was zero because YTN2 did not have this mutation. While YTN16 highly expressed Scarb2 gene with FPKM value of 54.31, VAF of mScarb2 in YTN16 was only 9%, suggesting that most of them were wild-type Scarb2. Because VAF of mZfp106 was 1.0 in YTN2 and YTN16, FPKM values reflected the level of mZfp106 expression, and they were comparable between YTN2 than YTN16. The difference in mRNA expression of neoantigen might explain why only mCdt1 peptide was identified by MHC class I ligandome analysis (Figure 5).

### 3.6. Determining the Optimal Epitope for mCdt1-Reactive CD8^+^ T Cells

**Figure 6:**
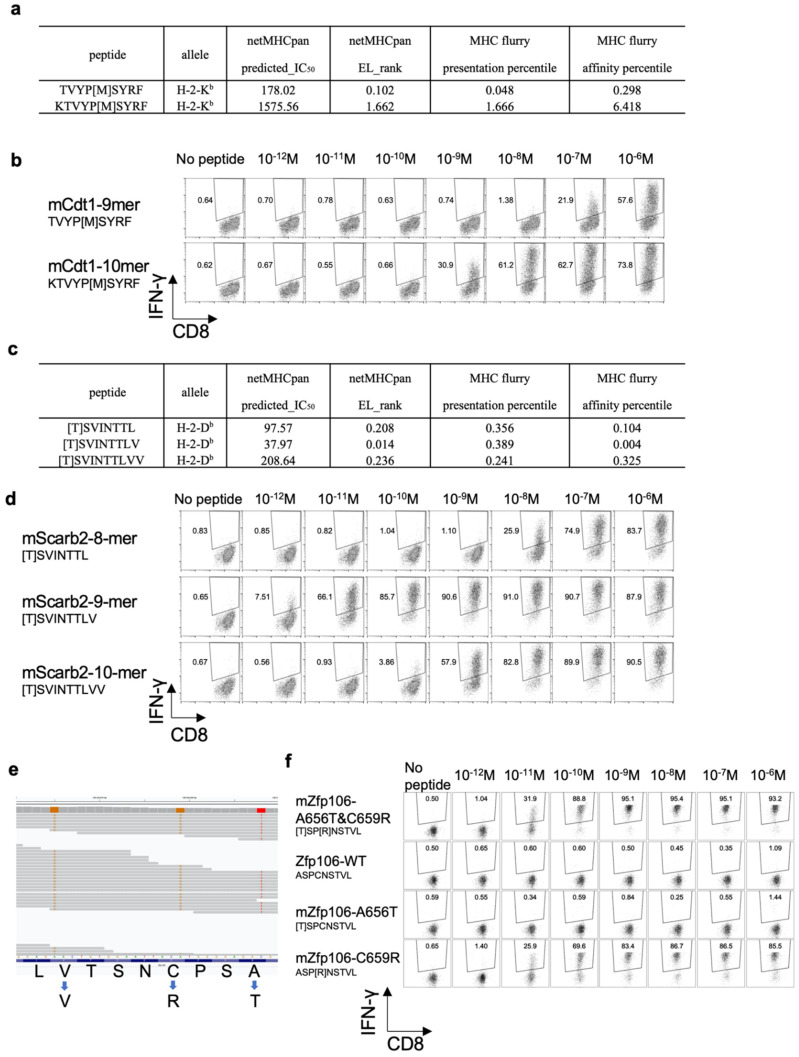
Functional affinities of three neoantigens: (**a**) MHC class I binding prediction values of 9-mer and 10-mer mCdt1 epitopes. (**b**) mCdt1-reactive CD8^+^ T cells were stimulated with 9-mer or 10-mer mCdt1 peptides at the indicated concentrations. IFN-γ production was evaluated by intracellular cytokine staining. (**c**) MHC class I binding prediction values of 8-mer, 9-mer, and 10-mer mScarb2 peptides. (**d**) mScarb2-reactive CD8^+^ T cells were stimulated with 8-mer, 9-mer, or 10-mer mScarb2 peptides at the indicated concentrations. IFN-γ production was evaluated by intracellular cytokine staining. (**e**) An IGV screenshot of the WES reads of the mZfp106 gene. (**f**) mZfp106-reactive CD8^+^ T cells were stimulated at the indicated concentrations with mZfp106 A656T&C659R, wild-type, A656T or C659R peptides. IFN-γ production was evaluated by intracellular cytokine staining.

The 10-mer mCdt1 peptide, KTVYP[M]SYRF, had not been identified by the in silico prediction of MHC class I binding. The predicted IC_50_ value of the 10-mer mCdt1 was 1575.56 nM, while that of the 9-mer mCdt1 TVYP[M]SYRF was 178.02 nM. In addition, the EL rank in NetMHCpan, as well as the presentation percentile and affinity percentile of MHCflurry indicated better scores for the 9-mer mCdt1 TVYP[M]SYRF than for the 10-mer peptide (Figure 6a). Because ligandome analysis indicated that tumor cells presented the 10-mer, not the 9-mer, mCdt1 peptide on their H-2K^b^ molecules, we compared the reactivity of mCdt1-reactive CD8^+^ T cells to these 9-mer and 10-mer peptides. Consistent with the fact that both YTN2 and YTN16 cell lines harbored the mCdt1 mutation, mCdt1-reactive CD8^+^ T cells responded to YTN2, though to a lesser extent than to YTN16 (Figure 3a). Although they responded equally well to 9- and 10-mer peptides at 1 μg/mL, there was a difference in response at lower concentrations (Figure 6b). The lower limit of the amount of 9-mer mCdt1 peptide to stimulate mCdt1-reactive CD8^+^ T cells was 10^−7^M, whereas the 10-mer peptide induced IFN-γ production at only 10^−9^M. These results indicate that the 10-mer KTVYP[T]SYRF was the optimal epitope of mCdt1.

### 3.7. Optimal Epitope Peptide for mScarb2-Reactive CD8^+^ T Cells

In the case of mScarb2-reactive CD8^+^ T cells, three peptides, 8-, 9-, and 10-mers, were listed as candidate neoepitopes; the predicted IC_50_ of these was 97.6, 38.0, and 208.6 nM, respectively (Figure 6c). Unfortunately, ligandome analysis failed to detect any of these three peptides. Therefore, comparing the functional avidity of mScarb2-reactive CD8^+^ T cells found that they did not respond to wild type 8-mer Scarb2, but did respond to the 8-mer mScarb2 peptide at a concentration as low as 10^−8^ M (Figure 6d). In contrast, they responded to as little as 10^−11^ or 10^−12^ M of 9-mer mScarb2 peptide. They also responded to 10-mer mScarb2 at 10^−9^ M. These results suggest that the 9-mer peptide was the optimal epitope to stimulate mScarb2-reactive CD8^+^ T cells.

### 3.8. Proximal Variants That Alter the Peptide Sequence

For Zfp106, there were three proximal mutations, G1966A, T1975C, and T1989C. Of these, G1966A and T1975C resulted in amino acid mutations, A656T and C659R, while T1989C was synonymous. When WES reads were visualized by Integrative Genomics Viewer (IGV), both G1966A and T1975C mutations were found on the same reads (Figure 6e). Therefore, tumor cells produced mZfp106A656T&C659R [T]SP[R]NSTVL, but neither mZfp106-A656T [T]SPCNSTVL nor mZfp106-C659R ASP[R]NSTVL. As expected, the mZfp106-reactive CD8^+^ T cell line responded to mZfp106 A656T&C659R peptide at as little as 10^−11^ M (Figure 6f). Furthermore, although A656T [T]SPCNSTVL peptide did not stimulate mZfp106-reactive CD8^+^ T cells at all, the C659R ASP[R]NSTVL peptide stimulated IFN-γ production as well as the mZfp106 A656T&C659R peptide (Figure 6f). This suggests that C659R is critical for recognition by mZfp106-reactive TCR.

### 3.9. Neoantigen-Based Immunotherapy for YTN16 Tumors

**Figure 7:**
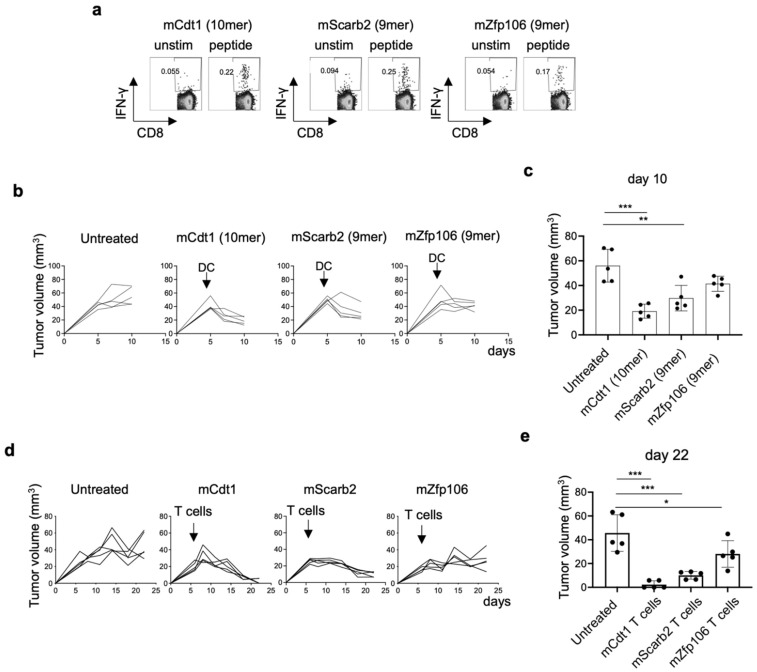
Anti-tumor effects of neoantigen-based immunotherapy: (**a**) Mice were vaccinated with neoepitope peptide-pulsed DCs twice biweekly. Two weeks after the second vaccine, the mice were sacrificed, splenocytes were stimulated with corresponding short peptides for 4 h, and IFN-γ production was evaluated by intracellular cytokine staining. (**b**) Mice (*n* = 5) were inoculated with 5 × 10^6^ YTN16. DCs pulsed with neoepitope peptides were injected subcutaneously 5 days after tumor inoculation. Tumor volumes were monitored every 2 or 3 days. (**c**) Tumor volumes on day 10 were shown. ** *p* < 0.01, *** *p* < 0.0001, one-way ANOVA with Dunnett’s test for multiple comparisons. (**d**) Mice (*n* = 5) were inoculated with 5 × 10^6^ YTN16. Six days later, 1 × 10^7^ neoantigen-reactive CD8^+^ T cells were injected intravenously. Tumor volumes were monitored every 2 or 3 days. (**e**) Tumor volumes on day 22 were shown. * *p* < 0.05, *** *p* < 0.0001, one-way ANOVA with Dunnett’s multiple comparison test.

As described above, we established three neoantigen-reactive CD8^+^ T cells and identified three neoepitopes (Figure 3). The 10-mer mCdt1 and the 9-mer mZfp106 expressed by both YTN2 and YTN16, and the 9-mer mScarb2 unique to YTN16. Therefore, we conducted neoantigen-based immunotherapy for YTN16 tumors. First, neoantigen-reactive CD8^+^ T cells were induced by DC vaccines. C57BL/6 mice were immunized with DCs pulsed with mCdt1, mScarb2, or mZfp106 peptides (Figure 7a). Spleen cells from immunized mice selectively produced IFN-γ in response to corresponding immunized peptides. Then, YTN16 tumor-bearing mice were therapeutically treated with mCdt1, mScarb2 or mZfp106 peptide-pulsed DCs (Figure 7b). Tumor growth was successfully inhibited by these neoantigen DC vaccines; however, the DC vaccine could not eradicate the tumors (Figure 7c). Next, we conducted an adoptive transfer of neoantigen-reactive CD8^+^ T cells. YTN16 tumor-bearing mice received 1 × 10^7^ of mCdt1-, mScrab2-, or mZfp106-reactive CD8^+^ T cells (Figure 7d). mCdt1-reactive CD8^+^ T cells eradicated YTN16 tumors. mScarb2-reactive CD8^+^ T cells inhibited YTN16 tumor growth and reduced the tumor volume. mZfp106-reactive CD8^+^ T cells also suppressed YTN16 tumor growth, though to a lesser extent with the other 2 CD8^+^ T cells (Figure 7e).

## 4. Discussion

In the present study, we investigate the immunological characteristics of two gastric cancer cell sublines, YTN2 and YTN16, derived from the same parental line. Both are transplantable into C57BL/6 mice, but YTN2 spontaneously regresses, whereas YTN16 grows progressively (Figure 1). We identified two neoepitopes in YTN2 and three in YTN16 tumors by NGS-based in silico prediction of MHC-binding peptides (Figure 2 and Figure 3). However, MHC class I ligandome analysis only detected one of these neoepitopes, mCdt1 (but of the correct length, see Figure 5). Furthermore, we carefully characterize the fine specificity of neoepitopes (Figure 6), enabling us to evaluate the anti-tumor activity of neoantigen-based immunotherapy (Figure 7).

Tran et al. described that targeting cancer neoantigens by T cells is the “final common pathway”, resulting in cancer regression elicited by various cancer immunotherapies [16]. Therefore, the identification of neoantigens is crucial for the development of immunotherapy and immunomonitoring [17]. To do so, tumor-specific missense mutations are identified by WES; RNA-Seq is also combined with this to indicate the abundance of mutated antigens. After identifying tumor-specific mutations, several in silico peptide prediction algorithms are applied to filter the neoantigen candidates in terms of their binding capacity to MHC molecules (https://www.iedb.org/ (accessed on 1 December 2021)). In addition, the identification of specific neoepitopes eluted from tumor MHC molecules through immunopeptidomics, or LC-MS/MS-based immunopeptidomics, confirms that the neoepitopes are processed and presented at the cell surface [18]. In this manner, we approach the identification of actual neoantigens (defined as recognized by T cells), which are derived from only a small fraction of all identified tumor-specific mutations. In the present study, immunopeptidomics analysis determined that the neoepitope recognized by mCdt1-reactive CD8^+^ T cells was a 10-mer peptide rather than the 9-mer peptide predicted by in silico algorithms (Figure 5).

Another caveat for in silico neoantigen prediction is genome phasing. In typical NGS, the raw sequencing reads are aligned to the human reference genome; somatic variants are identified by comparing the tumor read alignments to normal ones. The resulting somatic variants are then annotated to predict protein sequence changes and reported merely as a list of variants without distinguishing between variants on homologous chromosomes. If there is any other sequence variant proximal to a somatic variant of interest in the patient’s genome that differs from the human reference, the real amino acid sequence of the mutated peptide may alter the amino acid sequence of the resulting peptide [19]. This is indeed the case in mZfp106 of the YTN2 and YTN16 paired cancer subclones (Figure 6e). Three mutations were identified in mZfp106, namely, G1966A, T1975C, and T1989C. The former two were nonsynonymous mutations and the last was synonymous. A manual review of aligned reads with the IGV [20] confirmed that mZfp106 A656T and C659R were located on the same sequence read. As expected, mZfp106-reactive CD8^+^ T cells recognized mZfp106A656T&C659R [T]SP[R]NSTVL (Figure 6f). The current neoantigen prediction pipelines may overlook neoepitope peptides containing multiple proximal mutations or single nucleotide polymorphisms. In practice, then, an individual patient´s sequence should be confirmed by IGV before evaluating the MHC binding affinity.

Because mScarb2-reactive CD8^+^ T cells recognized YTN16, and mCdt1- and mZfp106-reactive CD8^+^ T cells responded to YTN2 and YTN16 cells in vitro and in vivo (Figure 3 and Figure 7), these cancer cells indeed presented these neoepitope peptide/MHC class I complexes on their surface. However, only mCdt1 peptide was identified by MHC class I ligandome analysis in YTN2, not in YTN16 (Figure 5). In addition, neoantigen-based immunotherapies targeting mCdt1 were more efficiently controlled the YTN16 tumors in DC vaccination and ACT therapy than targeting mScarb2 and mZfp106 (Figure 7). These results suggest that mCdt1 might be the dominant neoantigen in YTN2 and YTN16 tumors. These results also explain why YTN2 spontaneously regressed and YTN16 grew progressively in immune-competent mice, even though YTN16 has an additional neoantigen mScarb2. However, the hierarchy or dominance of neoantigens should be confirmed by knocking down the mCdt1 gene or reversing the mutation to a wild-type sequence in these cancer cells.

Although we did identify three neoepitopes, it is certainly possible that others may have been overlooked. Our in silico prediction algorithm focused on the MHC class I binding affinity of peptides and lacked any consideration of antigen processing and peptide transport that impacts the production of MHC-binding neoepitopes. Some computational methods incorporate an evaluation of antigen processing (e.g., NetChop [21]) and peptide transport (e.g., NetCTL [22]), but none of them is yet perfect for predicting functional neoantigens. There are also some weaknesses in neoantigen identification by immunopeptidomics. In the present study, immunopeptidomics analysis detected mCdt1 only from YTN2, but not YTN16. mZfp106 and mScarb2 were not detected at all. The sensitivity of MHC ligandome analysis remains insufficient thus far, and identifying neoantigens still requires confirming tumor-specific T cell responses. Analytical methods will undoubtedly be improved in the future.

## 5. Conclusions

Here, we immunologically characterized two gastric cancer cell sublines derived from the same parental line but with very different behaviors. We provided information on the efficacies of ICI treatment and neoantigen-specific CD8^+^ T cell responses. We successfully demonstrated the neoantigen-based immunotherapy-controlled tumor growth. Identification of neoantigens enables monitoring of the dynamics of anti-tumor immune responses and helps evaluate the effect of combination immunotherapies on tumor-specific immune responses. This information will contribute to the development of novel combination immunotherapies for gastric cancer.

## Figures and Tables

**Table 1:** Comparison of YTN2 and YTN16 in terms of neoantigens.

		YTN2	YTN16
mutation	mCdt1	T258M	T258M
	mScarb2	wild type	K426T
	mZfp106	A656T & C659R	A656T & C659R
gene expression (FPKM)	Cdt1	50.67	47.91
	Scarb2	29.73	54.31
	Zfp106	43.58	38.22
RNA variant allele frequency (VAF)	mCdt1	0.52	0.41
	mScarb2	0.00	0.09
	mZfp106	1.00	1.00
MHC ligandome	mCdt1	T258M KTVYP[M]SYRF	not detected
	mScarb2	not detected	not detected
	mZfp106	not detected	not detected

## Data Availability

Data are deposited on the Sequence Read Archive (SRA) database (accession number: SRR12072973-75, SRR15647456 and SRR 17087999) and the Gene Expression Omnibus (GEO) database (GSE146027 and GSE184092).

## Supplementary Materials

The following supporting information can be downloaded at: https://www.mdpi.com/article/10.3390/cancers14010106/s1, Figure S1: HPLC fractionation of peptides eluted from YTN16 cells. Table S1: Missense mutations detected in YTN2. Table S2: Missense mutations detected in YTN16. Table S3: Candidate neoepitope peptides. Table S4: Kd values of neoepitope peptides measured by MHC class I stabilization assay. Table S5: Mutations in YTN2, YTN16, B16F10, LLC1, and MC38.
