# Supplementary material for: Identification of Neoantigens in Two Murine Gastric Cancer Cell Lines Leading to the Neoantigen-Based Immunotherapy
Source: Cancers (Basel). 2021 Dec 27;14(1):106. doi: 10.3390/cancers14010106 (PMC8750027; doi:10.3390/cancers14010106)
Supplement: Supplementary file 1 [file cancers-14-00106-s001.zip › Supplementary_Figure_Table/Supplementary_Figure_S1_HPLC fractionation of peptides eluted from YTN16 cells.pdf]

Supplementary Figure S1

a

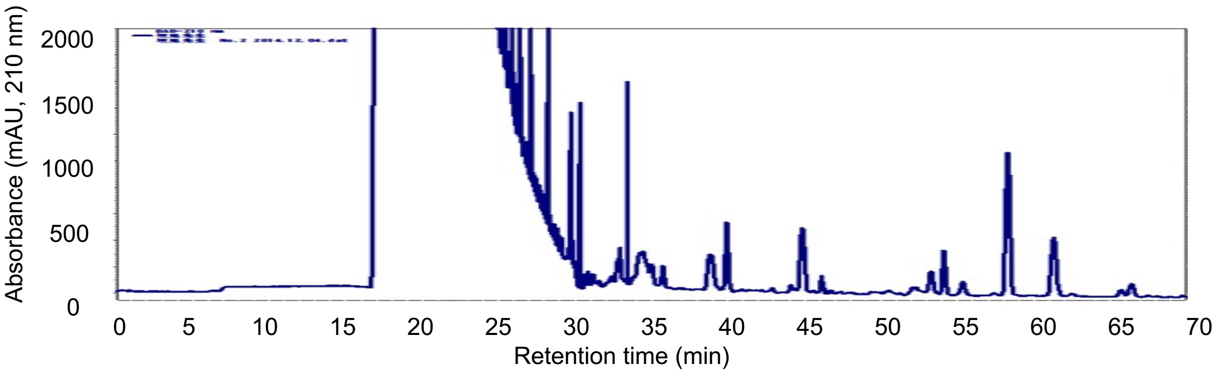

b

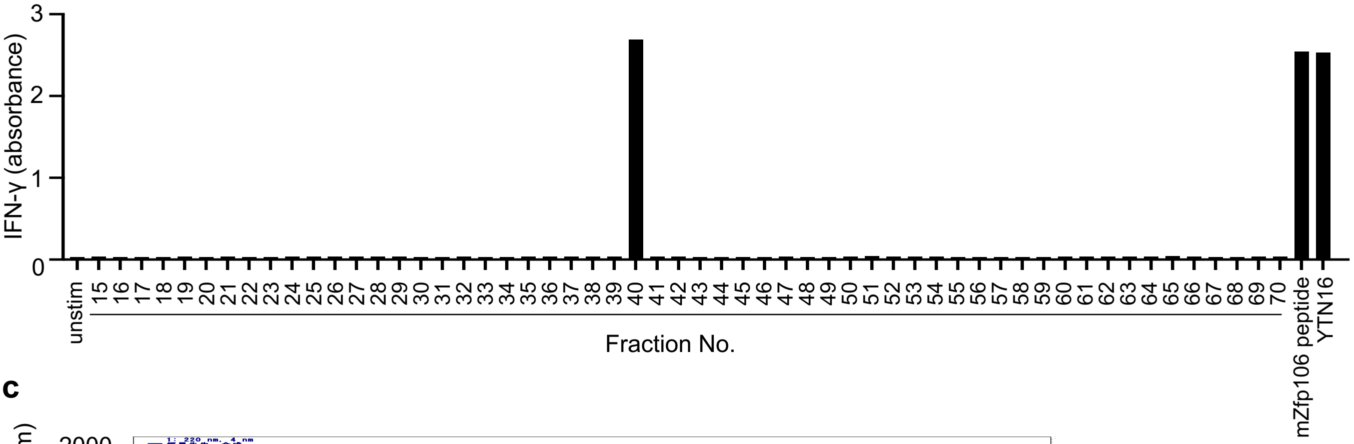

c

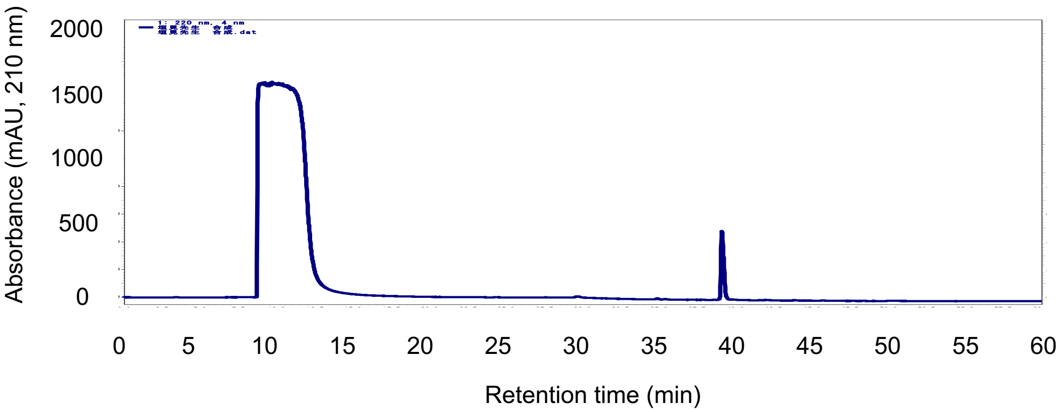

Supplementary Figure S1. HPLC fractionation of peptides eluted from YTN16 cells. **(a)** MHC class I-bound peptides were eluted from YTN16 by mild acid treatment and fractionated by reverse-phase HPLC. **(b)** Each fraction was assessed for stimulation of the mZfp106-reactive CD8^{+} T cell line. IFN-γ production was evaluated by ELISA. **(c)** Synthetic mZfp106 peptide was fractionated under the same conditions as **(a)**.
